# Exploring the antecedents of trust in electronic word-of-mouth platform: The perspective on gratification and positive emotion

**DOI:** 10.3389/fpsyg.2022.953232

**Published:** 2022-08-18

**Authors:** Xuemei Xie, Luyao Liu

**Affiliations:** School of Economics and Management, Beijing University of Posts and Telecommunications, Beijing, China

**Keywords:** e-wom platform, platform trust, gratification, positive emotion, S-O-R paradigm

## Abstract

Frequent human-media interaction *via* the electronic word-of-mouth (e-wom) platform, trust is acknowledged as an ongoing challenge. This study aimed to understand users' trust in the e-wom platform based on uses and gratifications theory and stimulus-organism-response (S-O-R) paradigm. Utilitarian gratification (perceived information quality and perceived privacy protection) was regarded as stimulus, social gratification (sense of social belonging and sense of self-esteem) and positive emotion as organism, and platform trust as response. Data was acquired from 268 users in China using a questionnaire survey, and the PLS-SEM was used to further analyze the results. The results indicated that there is a hierarchy relationship between types of gratifications. That is, utilitarian gratification is a premise of social gratification. Moreover, sense of self-esteem and positive emotion have a mediating effect between perceived information quality and platform trust. Sense of social belonging and positive emotion have a mediating effect between perceived privacy protection and platform trust. This study not only broadened trust between human and media, but also purposed a hierarchy relationship of different types of gratifications in e-wom platform.

## 1. Introduction

As new platform technologies are introduced, the topic of how to improve trust takes on a new dimension. The e-wom platform provides a new set of options for users to share and get information about products and services (Hu et al., 2019; Lin et al., 2020). Since its introduction in 2013, XIAOHONGSHU has grown to become China's most popular e-wom platform. On the XIAOHONGSHU platform, over 300 million people share their purchasing and life experiences, allowing other users to make better decisions. According to a recent report, the number of content creators on the XIAOHONGSHU platform reached 43 million by March 2021, and the number of notes surpassed 300 million. The number of monthly active users hit 282 million in October 2021. The above data reveal frequently increased human-media interaction on the e-wom platform. In the digital world, trust is the base between the platform and users, as well as the cornerstone of the platform's survival (Li and Lin, 2021). Therefore, it is meaningful to explore various antecedents that influence trust in the e-wom platform.

Prior studies mainly explored users' behaviors on e-wom information, such as adoption, engage, and spread. Some researchers explored why people adopt e-wom on social networking sites based on the attachment theory (Park et al., 2019). Attachment avoidance, attachment anxiety, and interaction effects are the antecedents of e-wom adoption. Some researchers investigated how former customers engage in e-wom (Azer and Ranaweera, 2022). Some researchers investigated various motivational factors that influence SNS users' e-wom intention (Chai et al., 2022). They found intrinsic motivational factors embracing altruism, self-efficacy, and self-expression universally influence SNS users' e-wom intention. However, empirical study on the antecedents of trust in the e-wom platform is still lacking.

Trust is not a rational cognitive process (Yuan et al., 2018). Emotional states play a significant role. In this study, we explored key factors influencing users' trust in the e-wom platform, which include various gratifications and positive emotion. Especially, based on users and gratifications (UG) theory, this study proposed utilitarian gratification (perceived information quality and perceived privacy protection) and social gratification (sense of social belonging and sense of self-esteem). Combined with stimulus-organism-response (S-O-R) paradigm, utilitarian gratification was considered as stimulus, social gratification, and positive emotion as organism, and platform trust as response. This study found that utilitarian gratification is a premise of social gratification. To some extent, social gratification and positive emotion have a mediating effect between utilitarian gratification and platform trust. This study extended UG theory and S-O-R paradigm in the context of the e-wom platform. We broadened e-wom research, especially regarding how users trust in the e-wom platform. a hierarchy relationship exists in different types of gratifications.

## 2. Theoretical foundation and hypotheses

### 2.1. Different types of gratifications

Uses and gratifications theory, was first created in the research field of mass media like radio, newspaper, television. Its goal is to figure out what motivates people to use certain types of media (Leung and Wei, 2000). Meanwhile, it examines why people choose one form of media over another in order to gratify a variety of needs (Katz et al., 1974). With rapid information system, today it is widely used in the research field of social media to better understand psychological state of users.

Present studies have classified gratifications obtained when using various social media. Some researchers investigated the determinants of continuance intention toward SNSs (Chang, 2018). They found perceived gratifications including information gratification, emotional gratification, and social gratification. Some researchers examined the effects of different gratifications on the continuance intention of using WeChat in China (Gan and Li, 2018). They identified four types of gratifications, namely hedonic gratification, social gratification, utilitarian gratification, and technology gratification. Some researchers determined the impact of gratifications and emotional state on users' adoption and continuance intention in Weibo (Gogan et al., 2016). They explored users' gratifications, namely hedonic gratification (entertaining value), social gratification (social value and social participation), and utilitarian gratification (information consumption, utilitarian value, and content participation). Some researchers examined continuance intention with live-streaming services based on UG theory (Hsu and Lin, 2021). They determined three gratifications, namely entertainment gratification, informativeness gratification, and sociability gratification. Some researchers examined the antecedents of grocery purchase behavior, and identified three types of gratification, including utilitarian gratification, hedonic gratification, and experiential gratification (Kim, 2021).

Present studies pay more attention on the parallel relationship among different gratifications, instead of seeking other relationship. To fill this research gap, this study intended to employ perspectives of hierarchy of needs to identify hierarchy relationship of different types of gratifications. Based on UG theory and characteristics of the e-wom platform, we purposed utilitarian gratification and social gratification. Utilitarian gratification includes perceived information quality and perceived privacy protection. Social gratification includes sense of social belonging and sense of self-esteem.

The definition of perceived information quality refers to the correctness and completeness of website information as it relates to products and transactions (Kim et al., 2008). Users' perceptions of information quality have a beneficial impact on their willingness to participate actively in platform communication and engagement (Lu et al., 2011). As a result, we predicted that if the e-wom platform continues to provide meaningful and useful information, users' sense of social belonging will improve dramatically. Therefore, we put forward the following hypothesis:

H1: Perceived information quality has a positive impact on sense of social belonging.

Self-concept is a comprehensive view formed by individuals' cognition of themselves in various aspects. Self-esteem is typically influenced by environmental cues, information (evaluation and expectation) from influential figures in societal structure, and sense of competence and efficiency that individuals have experienced (Brockner, 1988). When people create a sense of importance and worth for themselves, they develop self-esteem (Pan et al., 2012). Specific to the e-wom platform, on the one hand, when users contribute information about products or services, and others accept and recognize their knowledge, they believe that they are capable and valuable. Users, on the other hand, will feel appreciated and so boost their self-esteem if they collect knowledge on the platform and others actively assist them. According to a research paper from organization behavior (Zheng et al., 2017), knowledge sharing has a positive effect on organization-based self-esteem. Thus, when users on the e-wom platform provide or acquire high quality information, namely, they obtain information gratification, their sense of self-esteem would improve dramatically. Therefore, we put forward the following hypothesis:

H2: Perceived information quality has a positive impact on sense of self-esteem.

Individuals' activity in social networks with high and transparent sociability can be witnessed by numerous others, making it impossible to hide (Livingstone, 2008). Privacy issues may arise if too much information is shared and received by too many persons (Schwartz, 1968). Previous research has found that overly apparent privacy policies can deter users from sharing content (Brandtzæg et al., 2010). Privacy protection is an important aspect in the growth of social media, as it encourages social contact between users (Sapuppo and Seet, 2015). We propose that, because of the platform's openness, users not only communicate utilitarian knowledge but also emotional experiences. As a result, privacy protection is at the heart of social interaction. This study suggested that privacy protection creates a secure interactive environment, in which users strive to form social relationships with one another and increase their sense of social belonging after obtaining technology gratification. Thus, we put forward the following hypothesis:

H3: Perceived privacy protection has a positive impact on sense of social belonging.

Today, the number of weak-tie contacts has explored *via* online social media. Individual performance is divided into on-stage and off-stage, according to Goffman (2016). In weak ties, individuals are more likely to execute impression management, which means they are more likely to project a positive image in front of strangers (Luo and Cong, 2015). On the e-wom platform which characterized by weak ties, self-image management corresponds to “off-stage performance,” which is not visible to on-stage acquaintances. If the e-wom platform has adequate privacy protection, users prefer to perform self-image management on this platform, boosting individuals' sense of self-esteem. We predicted that the cornerstone of social gratification is privacy protection. Therefore, we purposed the following hypothesis:

H4: Perceived privacy protection has a positive impact on sense of self-esteem.

### 2.2. Social gratification and positive emotion

Positive emotion is linked to the satisfaction of a certain need, which is frequently accompanied by a good subjective experience, and can boost an individual's excitement and ability to participate in activities (Meng, 1989).

The augmentation of emotional value offered by self-expression and relationships with others through the platform, so that users are more emotionally linked to the platform, is referred to as a sense of social belonging (Hsu and Lin, 2016). Previous research has shown that using social networking sites can successfully lessen loneliness and increase pleasant emotions (Huang et al., 2016). Users on the e-wom platform have a sense of social belonging through social interaction such as commenting and messaging. Sense of social belonging can help people feel better. Therefore, we put forward the following hypothesis:

H5: Sense of social belonging has a positive impact on positive emotion.

Individual self-worth and importance are reflected in self-esteem, while positive emotion represents an individual's emotional condition, such as happiness. There is a relationship between cognition and emotion (Lavy and Littman-Ovadia, 2017). Thus, we suggest that individual self-esteem as a cognition can directly influence positive emotion. According to attribution theory (Heider, 1958), individuals' self-esteem can be attributed internally and externally. Users hint at their own taste and social standing by flaunting their wonderful lives, which is known as internal attribution. External attribution is that the e-wom platform has a mutual respect culture. Because the majority of users are young on the e-wom platform, have a high level of education and quality. Previous study in the field of psychology suggests that self-esteem has a positive influence on position emotion (Liu et al., 2018). Thus, we predicted that on the e-wom platform, sense of self-esteem can enhance positive emotional state. We put forward the following hypothesis:

H6: Sense of self-esteem has a positive impact on positive emotion.

### 2.3. Positive emotion and trust

Positive emotion refers to the emotion with pleasant feeling is generated when individuals are stimulated by internal and external environment, and meet their own needs (Fredrickson et al., 2008). According to the broaden-and-build theory, positive emotion extends the scope of people's attention and thought-action repertoires, and create enduring personal resources including social connections and intellectual resources, as well as flourish people's mental health (Fredrickson and Losada, 2005). In exchange for favorable expectations of another's intentions or actions, trust is a psychological state characterized by the willingness to tolerate vulnerability (Rousseau et al., 1998). Meanwhile, trust is defined by dynamic and situational social psychological phenomena, and it has a complicated link with people's psychology, with good feeling being one of the most essential components (Wang and Lian, 1998). The relationship between positive emotions and trust has been discovered through interpersonal trust studies. According to affect-as-information theory, individuals use their affective states as information when making decisions (Schwarz and Clore, 1983). Happiness and gratitude, both positive emotions, improve interpersonal trust, but rage, a negative emotion, diminishes interpersonal trust (Dunn and Schweitzer, 2005).

Further, this study extended trust from level of interpersonal relationship to human and media. When people make evaluative judgments about trust, specific emotions influence subsequent judgments (Dunn and Schweitzer, 2005). In other words, different emotions can provide information sources. Trust is not automatic, but based on positive emotions. Positive emotion can boost people's positive perceptions of their risk partner, leading to greater positive decision-making, including trust (Bless and Fiedler, 2006). We predicted that when users place their trust in the e-wom platform, positive emotion offers evidence about trust judgment. Therefore, we put forward the following hypothesis:

H7: Positive emotion has a positive impact on platform trust.

### 2.4. The stimulus-organism-response paradigm

Given that utilitarian gratification is a crucial stimulus for the platform trust, users' social gratification and positive emotion dominate their final trust decision (response). The justification for using the stimulus-organism-response (S-O-R) paradigm as the theoretical lens to examine how types of gratifications and positive emotion influence trust in the e-wom platform is as follow. First, prior researchers (Yuan et al., 2020, 2021) applied the S-O-R paradigm to predict users' attitude, such as loyalty in social media. Second, its theoretical justification of examining utilitarian gratifications as stimuli and its capability of evaluating the role that users' emotional perceptions (social gratification and positive emotion) to utilitarian gratification plays in users' trust in the e-wom platform.

S-O-R paradigm to specify mediating processes in an organism that transmit a stimulus to a response. The term organism refers to the internal processes and structures intervening between stimuli and the final responses emitted. The intervening processes and structures consist of perceptual, physiological, feeling, and thinking activities. Response pertains to psychological reactions such as attitudinal and behavioral reactions. According to the S-O-R paradigm, users' utilitarian gratification (stimulus) may affect users' emotional perceptions (social gratification and positive emotion) (organism), which in turn may influence users' trust in the platform (response).

Therefore, we proposed the following hypotheses:

H8a: Sense of social belonging and positive emotion play chain double-mediation effects on the relationship between perceived information quality and platform trust.
H8b: Sense of self-esteem and positive emotion play chain double-mediation effects on the relationship between perceived information quality and platform trust.
H9a: Sense of social belonging and positive emotion play chain double-mediation effects on the relationship between perceived privacy protection and platform trust.
H9b: Sense of self-esteem and positive emotion play chain double-mediation effects on the relationship between perceived privacy protection and platform trust.

**Figure 1:**
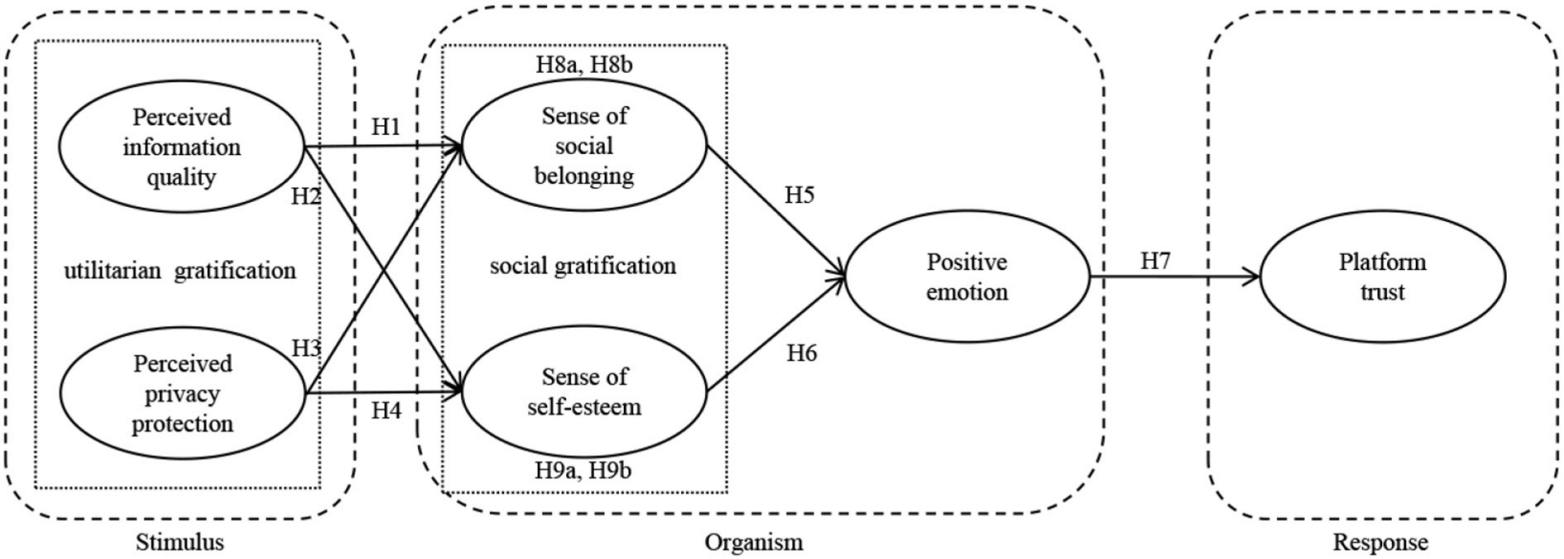
Theoretical model.

Theoretical model is as shown in Figure 1.

## 3. Research methodology

### 3.1. Measurement

Multiple items are used to measure all constructs, which are gathered in the survey using a five-point Likert scale ranging from 1 (strongly disagree) to 5 (strongly agree). The constructs are mostly adapted from earlier studies, but have been modified to request data on the e-wom platform. To ensure content validity, we invite experts to modify these items and users of the e-wom platform to do a pre-test.

The measurement of perceived information quality is mainly referenced to Kim et al. (2008). The measurement of perceived privacy protection is mainly referenced to Kim et al. (2008). The measurement of sense of social belonging is mainly referenced to Lin (2008). The measurement of sense of self-esteem is mainly referenced to Rosenberg (1965). The measurement of positive emotion is mainly referenced to Fredrickson (2013). Finally, the measurement of platform trust is mainly referenced to Suh and Han (2003). Items and sources are as shown in Table 1.

**Table 1:** Item and source.

**Construct**		**Item**	**References**
Perceived information quality (PIQ)	PIQ1	I think the word-of-mouth information within this platform is reliable	Kim et al., 2008
	PIQ2	I think the word-of-mouth information within this platform is useful.	
	PIQ3	I think the word-of-mouth information within this platform is of high quality.	
Perceived privacy protection (PPP)	PPP1	I think this platform will not use my personal information for other purposes without my authorization.	Kim et al., 2008
	PPP2	I think this platform will not share my personal information with other entities without my authorization.	
	PPP3	I think unauthorized persons have not access to my personal information.	
Sense of social belonging (SSB)	SB1	I enjoy being a member of this platform.	Lin, 2008
	SB2	I am very committed to this platform.	
	SB3	I enjoy this platform that has a high level of morale.	
	SB4	I feel a strong sense of social belonging to this platform.	
Sense of self-esteem (SSE)	SE1	I more recognize myself in the process of using the e-wom platform.	Rosenberg, 1965
	SE2	I feel good about myself in the process of using the e-wom platform.	
	SE3	I earn respect for myself in the process of using the e-wom platform.	
Positive emotion (PE)	PE1	I feel happy in the process of using the e-wom platform.	Fredrickson, 2013
	PE2	I feel positive in the process of using the e-wom platform.	
	PE3	I feel interested in the process of using the e-wom platform.	
Platform trust (PT)	TR1	I believe that this platform keeps its promises and commitment.	Suh and Han, 2003
	TR2	I believe that this platform meets users' expectations.	
	TR3	I believe that this platform keeps users' best interests in minds.	

### 3.2. Data collection and sample description

We disseminate the online questionnaire using Wenjuanxing, a professional online questionnaire platform. Only respondents who had used the e-wom platform before the poll were eligible to participate, in order to confirm that respondents matched the research purposes. Finally, we collected 327 surveys, however some were discarded because respondents failed the attention check questions or replied the same answer for all items, leaving 268 valid questionnaires with a valid response rate of 81.96.

Table 2 shows the demographic information characteristics of the valid samples. Obviously, the demographic characteristics of the respondents match the users of the e-wom platform.

**Table 2:** Demographic of respondents (*N* = 268).

**Demographic variable**	**Frequency**	**Percentage**
**Gender**		
Male	136	50.75
Female	132	49.25
**Age**		
18–25 years old	135	50.37
26–35 years old	123	45.90
36–45 years old	10	3.73
**Education**		
College and bachelor's degree	124	46.27
Master's degree	114	42.54
Doctor's degree	30	11.20
**Use frequency**		
Once a day or more	118	44.03
2–3 times a week	83	30.97
Once a week	11	4.10
2–3 times a month	28	10.45
Once a month or less	28	10.45

### 3.3. Data analysis

This study used partial least squares structural equation modeling (PLS-SEM) using Smart PLS 3.3 and its associated techniques, including the PLS algorithm and bootstrapping. In recent years, the number of articles published using PLS-SEM has increased significantly in contrast to covariance-based structural equation modeling (CB-SEM). Meanwhile, this study used SPSS software to perform several tests, such as descriptive and Harman's single-factor tests, which resulted in a 37.80% variation, which is less than acceptable threshold 50% (Podsakoff et al., 2003).

## 4. Results analysis

There are two stages to PLS-SEM analysis: measurement model and structural mode (Hair et al., 2018).

### 4.1. Measurement model

In this part, we accessed reliability and validity of measurement model. As shown in Table 3, regarding the reliability of the construct, the Cronbach's α of all constructs ranged between 0.830 and 0.927, which is above the acceptable threshold of 0.7 (Hair et al., 2018). The values of composite reliability (CR) ranged between 0.898 and 0.954, meeting criteria of 0.7 (Hair et al., 2018), which indicated adequate reliability. The convergent validity is assessed using two criteria, (1) standardized factor loadings of all items should exceed 0.7, and (2) the average variance extracted (AVE) of each construct needs to exceed the benchmark 0.5. As shown in Table 3, all items' factor loadings above the 0.7 threshold, and all the AVEs are above the benchmark value of 0.5. Thus, both conditions for convergent validity are adequate (Hair et al., 2018).

**Table 3:** Measurement model.

**Construct**	**Item**	**Loading**	**Cronbach's alpha**	**CR**	**AVE**
PIQ	PIQ1	0.853	0.831	0.898	0.745
	PIQ2	0.840			
	PIQ3	0.896			
PPP	PPP1	0.906	0.900	0.937	0.833
	PPP2	0.933			
	PPP3	0.899			
SSB	SSB1	0.845	0.887	0.922	0.746
	SSB2	0.880			
	SSB3	0.881			
	SSB4	0.849			
SSE	SSE1	0.939	0.927	0.954	0.873
	SSE2	0.947			
	SSE3	0.917			
PE	PE1	0.898	0.834	0.900	0.749
	PE2	0.862			
	PE3	0.836			
PT	PT1	0.882	0.830	0.898	0.746
	PT2	0.860			
	PT3	0.849			

To assess the discriminant validity, we used Fornell and Larcker's criteria (Fornell and Larcker, 1981). The square root of AVE of a construct needs to be greater than the correlation between the construct and other construct in this model. As shown in Table 4, the above criteria was clearly met.

**Table 4:** Analysis of discriminant validity.

**Construct**	**PIQ**	**PPP**	**PT**	**PE**	**SSE**	**SSB**
PIQ	0.863					
PPP	0.220	0.912				
PT	0.463	0.410	0.864			
PE	0.403	0.160	0.418	0.866		
SSE	0.289	0.214	0.381	0.554	0.934	
SSB	0.232	0.246	0.330	0.562	0.628	0.864

### 4.2. Structural model

To assess the structural model, path coefficients, coefficient of determination (*R*^2^), and cross-validated redundance (*Q*^2^) were used (Hair et al., 2018). Specifically, we employed bootstrapping for the path-coefficient calculation, the PLS algorithm for the *R*^2^ calculation, and blindfolding for the *Q*^2^ calculation. The *R*^2^ value evaluates the proposed model's predictive power and reflects the contribution of each construct. The *R*^2^ value ranges between 0 and 1, where valued of 0.20, 0.50, and 0.75 indicate weak, moderate, and substantial effects, respectively (Hair et al., 2018). The *Q*^2^ valued higher than zero are meaningful and that values of 0, 0.25, and 0.5 indicate small, medium, and large effects, respectively, representing the predictive accuracy of the model. This study used bootstrapping to test the path coefficients of the structural model. The results indicated that, based on the acceptance criterion (*t*-value > 1.96, *p*-value < 0.05).

**Figure 2:**
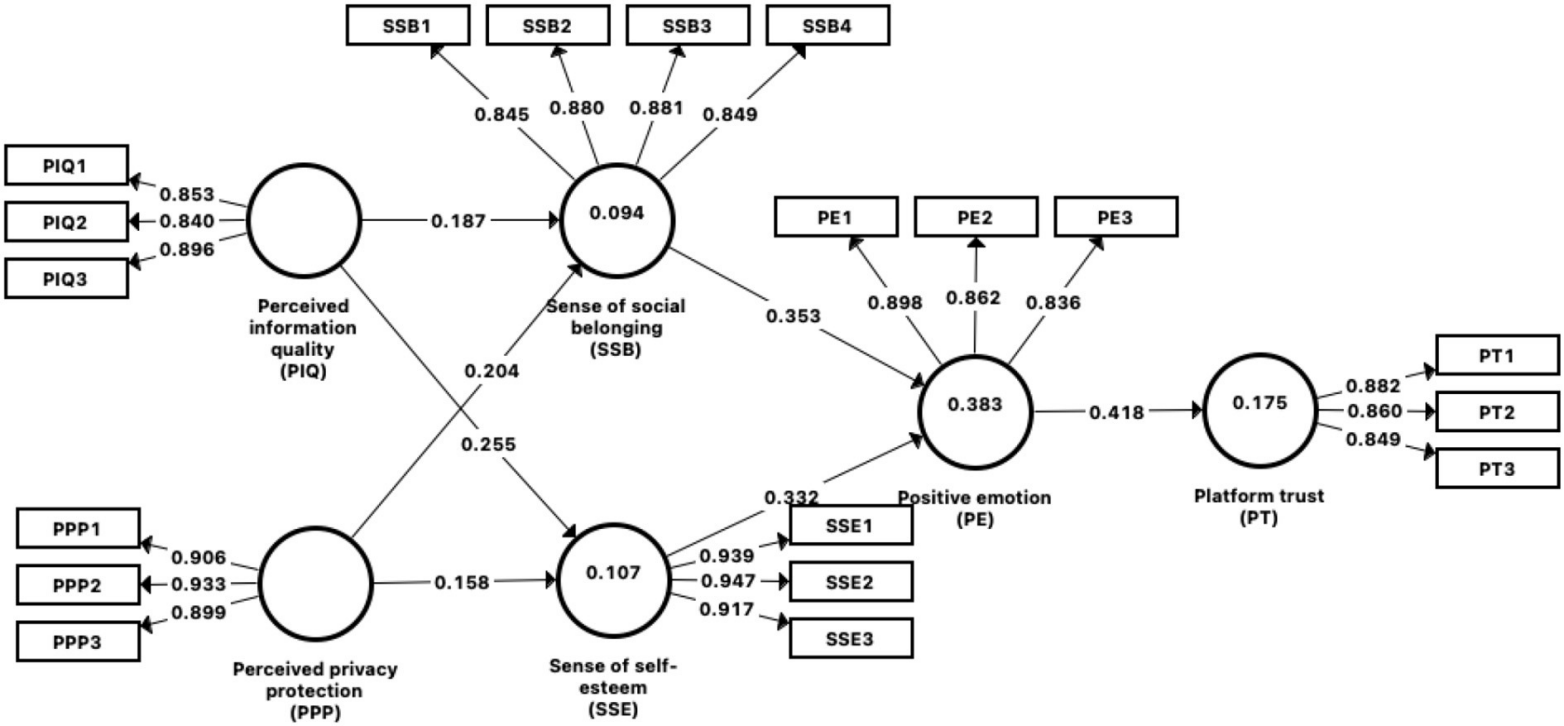
Structural model results.

The results are shown in Table 5 and Figure 2. The significant role of PIQ in driving SSB and SSE, with path coefficients of (*β* = 0.187, *t* = 2.581, *p* = 0.010) and (*β* = 0.255, *t* = 3.646, *p* < 0.001). Therefore, H1 and H2 are supported. PPP also plays a significant role in influencing SSB and SSE, with path coefficients of (*β* = 0.204, *t* = 3.426, *p* = 0.001) and (*β* = 0.158, *t* = 2.625, *p* = 0.009). Therefore, H3 and H4 are supported. SSB has a significant effect on PE (*β* = 0.353, *t* = 6.580, *p* < 0.001). Therefore, H5 is supported. SSE significantly affects effect on PE (*β* = 0.332, *t* = 6.164, *p* < 0.001). Therefore, H6 is supported. PE has a significant effect on PT (*β* = 0.418, *t* = 5.943, *p* < 0.001).

**Table 5:** Hypothesis testing and strength of the model.

**Hypothesis**	**Path**	**Path coefficient**	**Mean**	**SD**	***t*-value**	***p*-value**	**Decision**
**Direct effect**							
H1	PIQ ->SSB	0.187	0.193	0.073	2.581*	0.008	Supported
H2	PIQ ->SSE	0.255	0.258	0.070	3.646*	0.000	Supported
H3	PPP ->SSB	0.204	0.207	0.060	3.426*	0.001	Supported
H4	PPP ->SSE	0.158	0.161	0.060	2.625*	0.008	Supported
H5	SSB ->PE	0.353	0.357	0.054	6.580*	0.000	Supported
H6	SSE ->PE	0.332	0.330	0.054	6.164*	0.000	Supported
H7	PE ->PT	0.418	0.424	0.070	5.943*	0.000	Supported
**Indirect effect**							
H8a	PIQ ->SSB ->PE ->PT	0.028	0.030	0.015	1.837	0.065	Not supported
H8b	PIQ ->SSE ->PE ->PT	0.035	0.037	0.016	2.200*	0.026	Supported
H9a	PPP ->SSB ->PE ->PT	0.030	0.032	0.013	2.371*	0.018	Supported
H9b	PPP ->SSE ->PE ->PT	0.028	0.022	0.011	1.911	0.055	Not supported

*t−value>1.96(p < 0.05).

*R^2^ (SSB) = 0.094, R^2^ (SSE) = 0.107, R^2^ (PE) = 0.383, and R^2^ (PT) = 0.175*.

*Q^2^ (SSB) = 0.067, Q^2^ (SSE) = 0.09, Q^2^ (PE) = 0.277, and Q^2^ (PT) = 0.125*.

### 4.3. Mediation model

To investigate the mediating effect of SSB, SSE, and PE, the bootstrapping method was employed to estimate the indirect effect. As shown in Table 5, SSE and PE play a significant role between the relationship between PIQ and PT (*β* = 0.035, *t* = 2.231, *p* = 0.026); therefore, H8b is supported. SSB and PE play a significant role between the relationship between PPP and PT (*β* = 0.030, *t* = 2.374, *p* = 0.018). Therefore, H9a is supported. Finally, H8a (*β* = 0.028, *t* = 1.837, *p* = 0.065) and H9b (*β* = 0.022, *t* = 1.911, *p* = 0.055) are not supported. Because H8a and H9b not meet the criterion (*t*-value > 1.96, *p*-value < 0.05).

**Figure 3:**
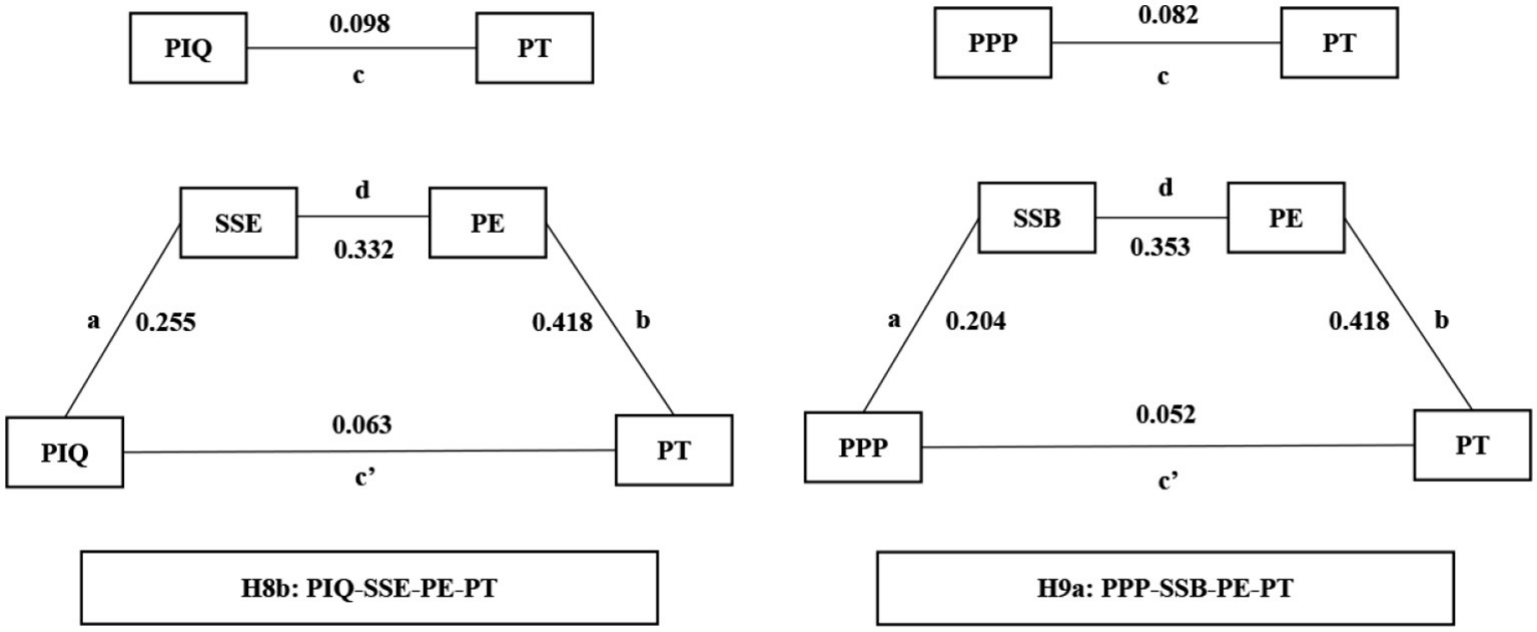
Mediation model results.

Then, in PLS-SEM, researchers generally calculate the strength of mediating effect. The variance accounted for (VAF) formula was employed (Hair et al., 2016). VAF = Indirect effect/Total effect, where Total effect = Indirect effect + Direct effect. VAF values of <20, 20–80, and >80% represent to no mediation, partial mediation, and full mediation, respectively (Hair et al., 2016). According to the above analysis, H8b and H9b are supported. Thus, this study calculated the strength of H8b and H9b. As shown in Figure 3, VAF values are 35.71 and 36.59%, respectively, which falls in the range between 20 and 80%, thus considered partial mediation. As shown in Figures 3, a, b, c, c′ and d represent path coefficient, a*b*d = indirect effect, c = total effect and c′ = direct effect.

## 5. Conclusion

The continued advancement of UGC and social media opens up new opportunities for both researchers and managers. The e-wom platform, as a popular information-exchange channel, requires immediate attention. Based on UG theory and S-O-R paradigm, this study investigated users' trust in the e-wom platform, especially how utilitarian gratification (perceived information quality and perceived privacy protection) affect platform trust through social gratification (sense of social belonging and sense of self-esteem) and positive emotion. The hypotheses mentioned are supported by empirical evidence from 268 valid questionnaires. This study offers a theoretical framework for increasing users' trust in the e-wom platform.

### 5.1. Findings

First, positive emotion has a positive impact on platform trust. This is in line with research in the field of interpersonal trust, which shows that positive emotion helps to improve trust (Lount, 2010). When people use the e-wom platform, positive feeling displays their happiness. Users create social friendships through commenting and texting, and locate like-minded people through communities and organizations in the platform. These are all positive emotion generators. Positive emotions, such as happiness and joy, are experienced while using the e-wom platform, which increases users' trust in this platform.

Second, as previous studies shown, sense of social belonging and sense of self-esteem are determinants of positive emotion (Yagil and Medler-Liraz, 2017). This study found that social gratification (sense of social belonging and sense of self-esteem) positively influences positive emotion. In comparison to reality social circles and We-chat moments, which are made up of strong relationships, the e-wom platform is made up of weak social ties. On the e-wom platform, users are more inclined to share their inner thoughts or sentiments, show their self-image, and release the pressures of reality. Users, for example, can meet like-minded sharers and feel a sense of social connection through information on this platform. Furthermore, individuals share things that they would not dare to exhibit in public, generate specific images, and get recognition and respect from others. As a result, when users perceive sense of social belonging and self-esteem, they will be in pleasant emotional states.

Third, utilitarian gratification (perceived information quality and perceived privacy protection) positively influence social gratification (sense of social belonging and sense of self-esteem). This finding is not surprising. According to hierarchy of needs theory, personal needs progress from a low to a high condition. It follows general law of growth of personal needs to some extent. Primary function of the e-wom platform is to offer e-wom information and privacy protection. Users will have regular conversation and engagement with other users once this platform becomes an efficient information reference source. Users will show their lives, share items and services, and hint at their own taste and social position when they perceive high privacy protection. Therefore, after obtaining utilitarian gratification, users further seek for social gratification on this platform.

Finally, this study figured out the mediating effects of social gratification and positive emotion. Especially, sense of self-esteem and positive emotion play a significant role in the relationship between perceived information quality and platform trust. Sense of social belonging and positive emotion play a significant role in the relationship between perceived privacy protection and platform trust. When users perceive more information quality, sense of self-esteem and positive emotion will make them trust this platform. When this platform provides good privacy protection, it is easy for user to trust this platform once obtaining sense of social belonging and positive emotion.

### 5.2. Implications for research

This study contributes to social media trust research. First, prior researches primarily focused on a single component when predicting platform trust, or used platform trust as a predictor to investigate the impact on user behavior. This study investigated the dynamic mechanism of platform trust development. It expanded on the existing study framework and brought a new research perspective to the topic of diverse types of social media trust. This study expanded the relationship between self-concept and emotional states from a micro perspective.

Second, this study broadened the application of UG theory and S-O-R paradigm. This study purposed gratifications of the e-wom platform, namely utilitarian gratification and social gratification. According to S-O-R paradigm, this study treated utilitarian gratification as stimulus, social gratification and positive emotion as organism, and platform trust as response. Although there are considerable researches into users' gratification in various types of social media, few studies looked at the interaction among different types of gratifications. According to this study, different types of gratifications play different roles. Utilitarian gratification is a predictor of social gratification. This study provided a theoretical framework of hierarchical structure among types of gratifications in social media usage.

Third, we don't understand the importance of positive emotion in social media well-enough. This study investigated the impact of positive emotion on platform trust, and found that social gratification influences positive emotion. This study applied positive emotion into social media trust. This study illustrated that positive emotion plays a significant role in the domain of human-media trust.

Last but not least, this study provided a deeper understanding of the process and mechanisms that lead from utilitarian gratification to platform trust. We not only found the essential reason of e-wom platform trust, but also other factors (social gratification and positive emotion) that can contribute to it. Understanding the appearance of trust is not the purpose of this study. In-depth research of mediating effects is a key step in studying the complex variable of trust, and it is an important part of promoting trust in human-media interaction.

### 5.3. Implications for practice

This study offered a new insight into how to boost trust in the e-wom platform. How to make an appealing e-wom platform that inspires users to trust it. These findings can be used to create a more persuasive and trustworthy e-wom platform. The e-wom platform provides numerous advantages for both individuals and businesses. These advantages, however, will not be achieved unless the e-wom platform establishes user trust.

First, positive emotion among users is a good predictor of platform trust. As a result, e-wom platform service providers should review user experience on a regular basis. Positive emotion is predicted by social gratification (sense of social belonging and sense of self-esteem). Service providers should organize social online activities on a regular basis to broaden users' social habits and improve user connection. Differentiating titles or interface features based on user level lets users feel valued and respected.

Then, this study discovered that utilitarian gratification is a predictor of social gratification. Therefore, service providers should improve the service quality to further promote users' social interaction within the platform. To limit the homogenization of content received by consumers under the recommendation algorithm. Content filtering technology and trusted AI technology should be used. Constantly enhancing user interface experience to improve users' sense of the effectiveness of privacy protection, such as setting up privacy policy reading links and official privacy protection push notifications.

Especially, social gratification and positive emotion have different mediating effects between utilitarian gratification and platform trust. Therefore, the platform should find the right positioning and provide different types of services for users. If the e-wom platform focuses on high-quality information, it should pay attention on providing a respectful environment. When the e-wom platform is good at protecting users' privacy, it should place particular emphasis on creating a lively social atmosphere.

### 5.4. Limitation and future studies

There are several limitations that need to be considered. First, because this study focuses on the e-wom platform in China, the findings may be confined to Chinese users. Future studies should increase the universality of the research model by covering a broader survey population. Second, the majority of the respondents in this study are young users. Despite the fact that the sample represents the majority of users in China, there may be differences for users of different ages. To investigate the differences in gratifications, future studies should consider using age and gender as moderators. Third, in this study, duration of collecting data is relatively concentrated. Future studies should adopt a longitudinal research approach to explore the relationships among gratification, emotion and trust.

## Data availability statement

The original contributions presented in the study are included in the article/supplementary material, further inquiries can be directed to the corresponding author/s.

## Ethics statement

The studies involving human participants were reviewed and approved by Beijing University of Posts and Telecommunications. The patients/participants provided their written informed consent to participate in this study.

## Author contributions

XX contributed to conception, design of the study, and supervision. LL contributed to investigation, visualization, and writing—original draft. All authors contributed to manuscript revision, read, and approved the submitted version.

## Conflict of interest

The authors declare that the research was conducted in the absence of any commercial or financial relationships that could be construed as a potential conflict of interest.

## Publisher's note

All claims expressed in this article are solely those of the authors and do not necessarily represent those of their affiliated organizations, or those of the publisher, the editors and the reviewers. Any product that may be evaluated in this article, or claim that may be made by its manufacturer, is not guaranteed or endorsed by the publisher.
